# Combining the best of both worlds: radical-based divergent total synthesis

**DOI:** 10.3762/bjoc.19.1

**Published:** 2023-01-02

**Authors:** Kyriaki Gennaiou, Antonios Kelesidis, Maria Kourgiantaki, Alexandros L Zografos

**Affiliations:** 1 Aristotle University of Thessaloniki, Department of Chemistry, Laboratory of Organic Chemistry, Thessaloniki, 54124, Greece

**Keywords:** biomimetic synthesis, cascades, common scaffold, hydrogen atom transfer, photoredox catalysis

## Abstract

A mature science, combining the art of the total synthesis of complex natural structures and the practicality of delivering highly diverged lead compounds for biological screening, is the constant aim of the organic chemistry community. Delivering natural lead compounds became easier during the last two decades, with the evolution of green chemistry and the concepts of atom economy and protecting-group-free synthesis dominating the field of total synthesis. In this new era, total synthesis is moving towards natural efficacy by utilizing both the biosynthetic knowledge of divergent synthesis and the latest developments in radical chemistry. This contemporary review highlights recent total syntheses that incorporate the best of both worlds.

## Introduction

Societal needs push sciences into new directions, as the urge for new pharmaceutical leads grows, in order to counteract global health challenges. Following this trend, total synthesis has been remodeled from the purely academic quest and display of human abilities to synthetically achieve natural complexity [1] to a modern science addressing the need for the supply of natural products and congeners for biological screening.

**Scheme 1:**
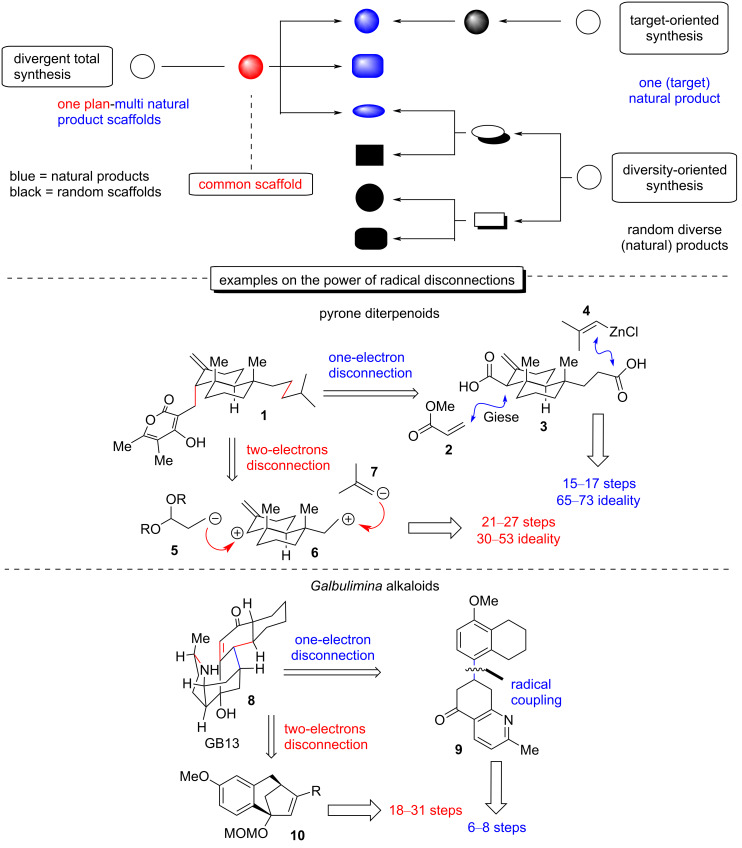
The power of radical retrosynthesis and the tactic of divergent total synthesis.

The era of scalability [2] in total synthesis prompts researchers in this field to make use of more direct retrosynthetic disconnections with the aid of “radical” retrosynthetic analysis, as the advancement in the area now allows to harness one-electron power in a highly chemoselective manner [3]. The development of persistent radicals [4] as synthons in chemical synthesis, coupled with the advancements in generating and manipulating transient radicals [5] as cross-coupling partners in an array of chemical reactions, gives access to a wide variety of “new” retrosynthetic disconnections. As radical disconnections are gaining ground, more sophisticated retrosyntheses of natural products are unlocked, enriching thus their synthetic scalability [6–7]. A direct comparison of a classic vs a radical approach highlights the complementarity and, more often than not, the superiority of the latter, which is proven in the number of steps and the overall yield, hence establishing it as highly appealing for further development (Scheme 1).

In order to identify the new pharmaceutical leads of tomorrow, drug discovery relies on the available chemical space rising from existing chemical libraries. But how big should this chemical space be, so as to actually address our needs? A general consensus has emerged, supporting that “it is not actually the library size but rather the library diversity in terms of molecular structure and function which is fundamental for a successful drug discovery” [8]. And this is where divergent total synthesis might help. Divergent total synthesis is an old but yet underdeveloped strategy, utilizing the conceptual advantages of biosynthetic routes that allow multi-target natural product synthesis through a unified synthetic plan [9–10]. Based on the logic of divergent synthesis, common synthetic scaffolds, which are regarded as the points of diversity of the synthetic plan, lie at the heart of retrosynthetic design. Radical disconnections on common scaffolds, in accordance to the trends of green chemistry [11] and the concepts of atom economy [12] and protecting-group-free synthesis [13], are gradually drawing more and more the interest of organic chemists as a sustainable way to deliver structurally diverse chemical libraries for biological screening. The current review is focusing on selected examples utilizing a radical-based divergent total synthesis approach, excluding electrochemical methods for generating radicals. An exhaustive review on radical total synthesis or divergent total synthesis lies beyond the scope of this review, and the readers are advised to refer to excellent reviews on these topics [6,10,14]. This review covers the years 2018–2022.

## Review

### Radical-based divergent synthesis

Commonly, the most successful divergent plans apply where the natural molecular complexity is rich. Not surprisingly, most of the divergent total syntheses carried out thus far are performed on terpenoid and alkaloid targets, utilizing common synthetic intermediates closely related to the biosynthetic origins of the family. On the other hand, radical retrosynthetic disconnections on common scaffolds are much less predictable and rarely similar due to the plethora of radical chemical transformations available nowadays.

**Figure 1:**
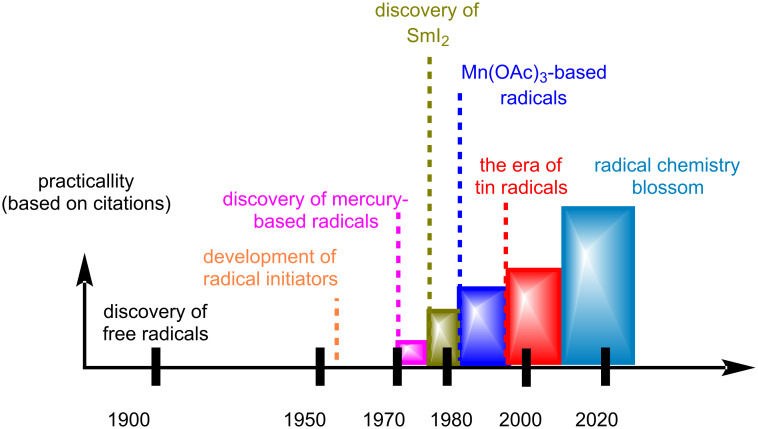
Evolution of radical chemistry for organic synthesis.

Although radicals stopped being confronted as “scientific curiosities” in the late 1960s, when radical initiators and organomercury reagents were developed as reliable reaction partners (Giese reaction) [15], it was not until the mid-1980s, at which point they appeared as key reaction players in total synthesis (Figure 1). The change in the perception that radicals cannot be selectively used took place with the introduction of tin hydrides in organic synthesis. Apart from the lower toxicity compared to organomercury reagents, the stability and longevity of tin-centered radicals allowed better propagation of radical chain reactions [16]. Based on their reactivity, major contributions in carbon-centered radical formation followed, consequently unlocking highly predictable intramolecular reactions, deoxygenation protocols (Barton–McCombie reaction) [17], etc. Other reagents that majorly contributed were samarium diiodide for the generation of radicals from carbonyl reduction [18] but also manganese(III) acetate as a convenient one-electron oxidant [19]. The next twenty years, the field continued to flourish mainly by way of the decipherment of hydrogen atom transfer (HAT) mechanisms, which led to the establishment of several reactions of transition metal hydrides (Fe, Co, Mn, etc) with alkenes (e.g., Mukaiyama hydration) [20].

The last decade saw the development of milder methods for generating carbon-centered radicals as the advancement of their reactivity in cross-coupling reactions, the concept of photoredox catalysis [21], and electrochemistry [22] all refuelled the field, allowing for more practical radical disconnections for total synthesis.

### Divergent synthesis of pyrone diterpenes

**Scheme 2:**
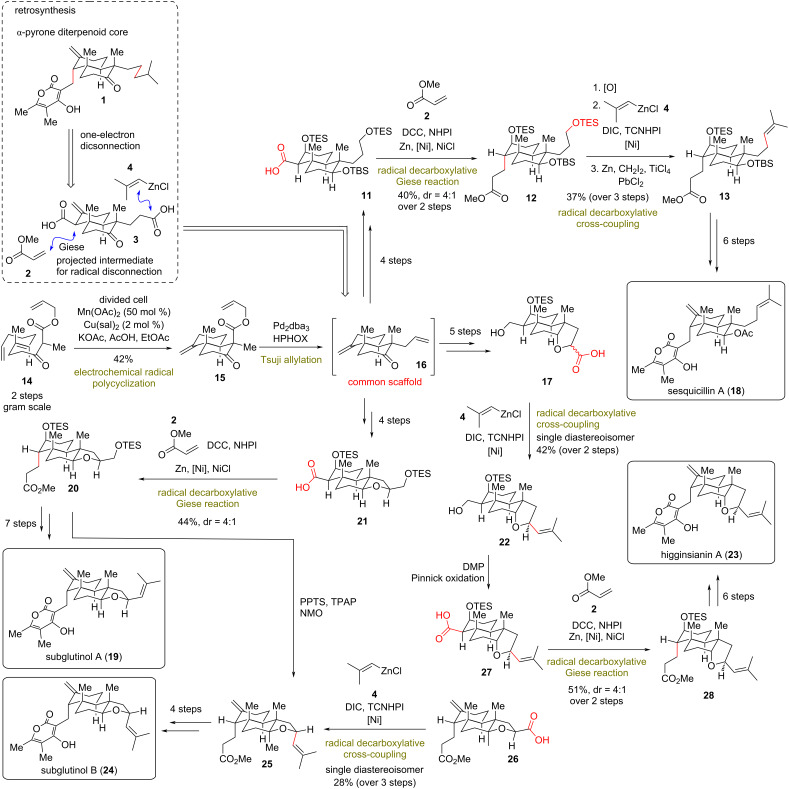
Divergent total synthesis of α-pyrone-diterpenoids (Baran).

**(Baran 2018)** [23]**:** The modestly sized family of pyrone diterpenes exhibits a wide range of bioactivities, ranging from immunosuppressive to hypertensive properties [24–26] depending on the subtle substituents in the periphery of a decalin core (Scheme 2). In 2018, the Baran group reported the divergent total synthesis of several pyrone diterpene natural products, relying solely on one-electron-based retrosynthesis. The group recognized the disadvantages that stemmed from prior 2-electron disconnections, namely the complicated C–C bond formations and the necessity for excessive functional group manipulations but also the unavailability of a unified divergent plan for this class of diterpenoids. As an alternative, they proposed nickel-mediated decarboxylative Giese reactions and decarboxylative radical zinc-mediated cross-coupling reactions of redox-active esters, established from previous works of the group [27–28], for the key C–C bonds of the diverse congeners. To this end, a hypothetical intermediate **3** was envisioned for their synthesis (Scheme 2). The synthetic variant of **3** was designed as the common scaffold **16**, bearing the appropriate substitution for sequential revelation of carboxylic acid moieties. The highly congested decalin core of common scaffold **16** was obtained by a modified electrochemical polycyclization of polyene **14** (prepared in two steps) in multigram quantities [23]. The reaction employed a divided cell with substoichiometric amounts of magnesium(II) acetate (0.5 equiv) and catalytic copper(II) 3,5-diisopropylsalicylate (0.02 equiv) to allow the redox radical cyclization of polyene in 42% yield. A Tsuji allylation using achiral H-PHOX followed to produce **16**, without being isolated. From this point of divergence, Baran’s group managed to reveal the requisite phthalimide carboxylates for each precursor of the diverse natural products and transformed it carrying out Giese reactions or nickel-catalyzed radical coupling to **13**, **20**, **25**, and **28**, few steps away from the total syntheses of sesquicillin A (**18**), subglutinols A and B (**19** and **24**) and higginsianin A (**23**, Scheme 2).

### Merged chemoenzymatic and radical synthesis of oxidized pyrone meroterpenoids

**Scheme 3:**
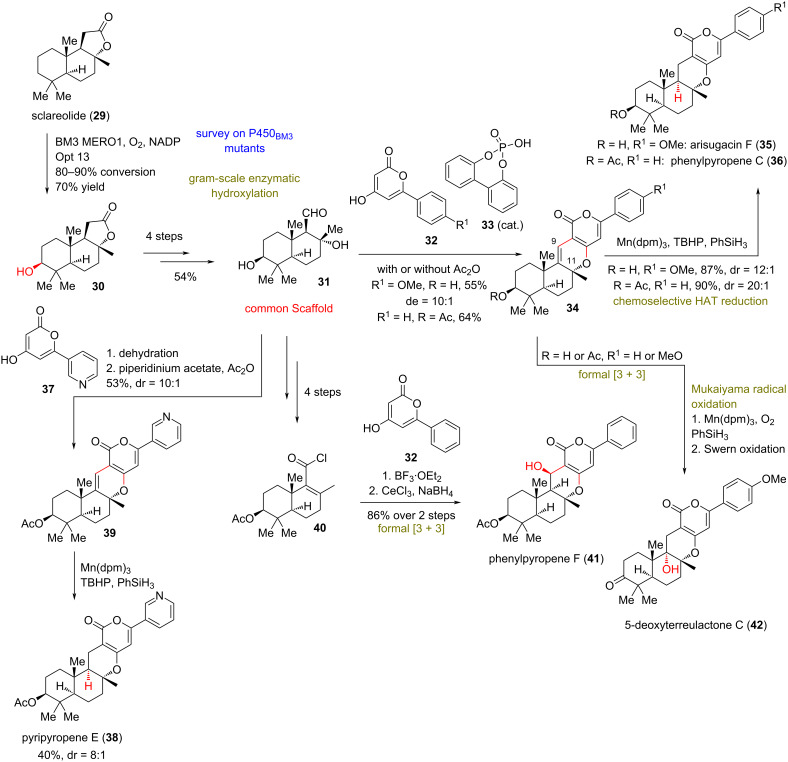
Divergent synthesis of pyrone diterpenoids by merged chemoenzymatic and radical synthesis (part I, Renata).

**Scheme 4:**
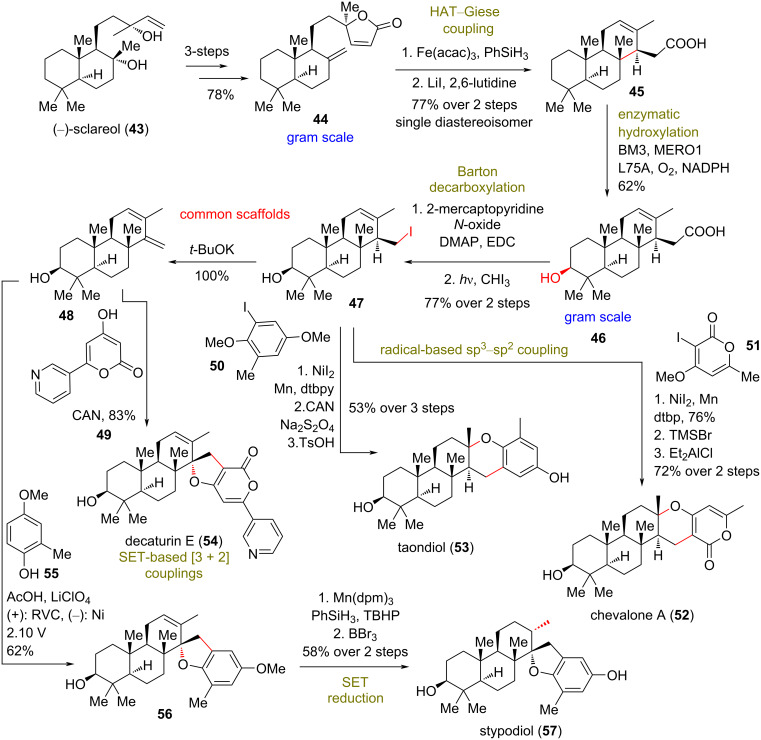
Divergent synthesis of pyrone diterpenoids by merged chemoenzymatic and radical synthesis (part II, Renata).

**(Renata 2020)** [29]**:** In 2020, a different approach was conceptualized by Renata’s group to access various oxidized members of pyrone meroterpenoids. The divergent plan of Renata’s group depended on the development of a highly chemoselective, chemoenzymatic 3-hydroxylation of sclareolide (**29**) and (−)-sclareol (**43**, Scheme 3 and Scheme 4). The group began by conducting a brief survey of several P450 BM3 mutants, deducing that the variant 1857 V328A (BM3 MERO1) was able to achieve high conversion of sclareolide (**29**) to the hydroxylated counterpart **30** in >95% yield. Based on this success, the group employed a radical disconnection approach of several 3-hydroxylated pyrone meroterpenoids on sclareolide (**29**). Key reaction of this strategy was the formal [3 + 3] cycloaddition, catalyzed by phosphoric acid **33**, followed by addition of a pyrone residue **32** to sclareolide-derived aldehyde **31**, which served as the common synthetic intermediate for the synthesis (Scheme 3) [30]. HAT reductions of the C9–C11 alkene followed to deliver arisugacin F (**35**), phenylpyropene C (**36**), pyripyropene E (**38**), and phenylpyropene F (**41**). The steric bulk of the manganese catalyst employed suppressed the undesired reaction with tetrasubstituted alkenes and led to the exclusive reaction of the desired trisubstituted alkene due to stabilization of the incipient radical at C9. Furthermore, HAT reduction served to only deliver the thermodynamic product of the *trans*-decalin. Similarly, the C9–C11 alkene can serve as an ideal handle to C11-hydroxylated products, such as **42**, through a Mukaiyama hydration [20] to furnish natural complexity.

A similar approach was devised for the synthesis of modified meroterpenoids chevalone A (**52**), taondiol (**53**), decaturin E (**54**), and stypodiol (**57**, Scheme 4). For this purpose, tricycle **45** was prepared from compound **44** in gram-scale quantities by HAT–Giese coupling, followed by reductive cleavage of the lactone moiety with LiI. Enzymatic hydroxylation by the BM3 MERO1 variant worked equally well to provide the 3-hydroxylated product **46**. Photochemical radical decarboxylation of the formed mercaptopyridine derivative and radical capture by iodoform led to common scaffolds **47** and diene **48** after subsequent elimination. Those molecules serve as templates for Ni-based radical-based sp^3^–sp^2^ coupling and single-electron transfer (SET)-based [3 + 2] coupling, respectively (Scheme 4). Initial attempts to realize the [3 + 2] radical coupling with CAN led to competitive oxidation of the C3 alcohol to the respective ketone. Increasing the equivalents of pyrone led to 83% of **54**. On the other hand, employment of the same conditions to phenol **55** resulted only in the oxidation of the phenol. A more controlled delivery of electrons was realized by applying an electrochemical method to provide the desired coupling towards **56** in 62% yield. Radical reduction by Mn(dpm)_3_ afforded stypodiol (**57**) after BBr_3_-mediated deprotection. Nickel-catalyzed coupling under a Weix procedure [31] was selected in order to elaborate the cores of taondiol (**53**) and chevalone A (**52**) as the radical cross-coupling employing redox-active esters of carboxylic acid **46** proved unsuccessful. The coupling was followed by an acid-catalyzed cyclization to yield the pyrone core of the natural products. The divergent plan described provided various meroterpenoids in 7–12 steps, comprising one of the most concise methods to attain this class of compounds, highlighting the power of merged biocatalytic and radical tactics [32].

### (+)-Yahazunol (**61**) and related meroterpenoids

**Scheme 5:**
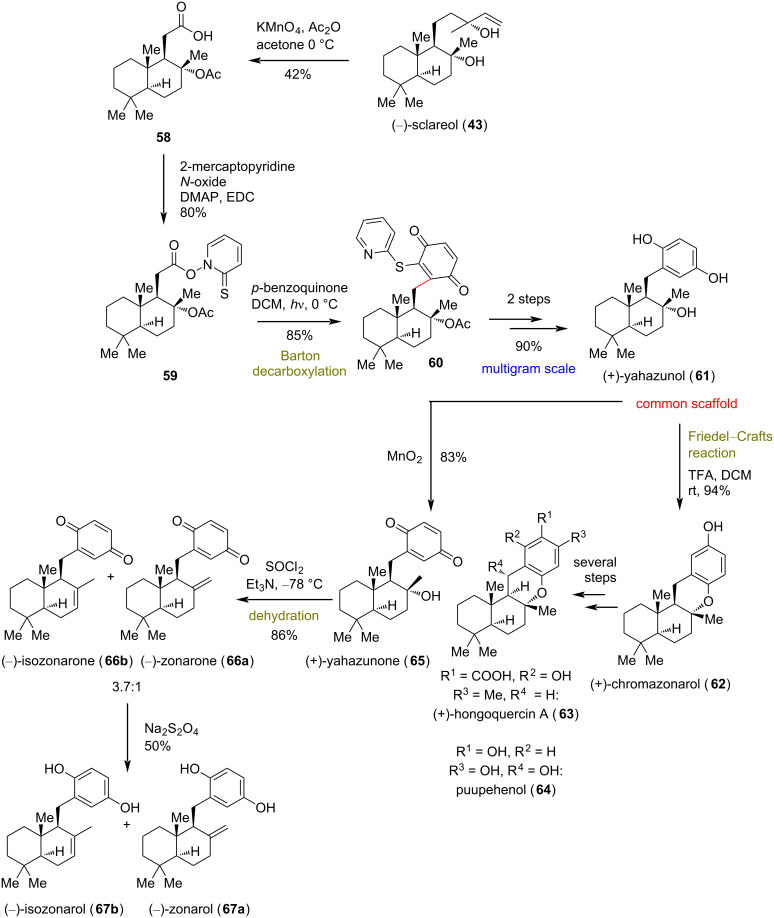
Divergent synthesis of drimane-type hydroquinone meroterpenoids (Li).

**(Li 2018)** [33]**:** In 2018, Li’s group reported a divergent plan for the synthesis of drimane-type hydroquinone meroterpenoids. This class of compounds possesses versatile bioactivities, ranging from anticancer and anti-HIV to antifungal properties, with minor modifications on the decoration of either the hydroquinone or the terpene part of the secondary metabolite [34]. The group applied a semisynthetic plan starting from (−)-sclareol (**43**) to access the common synthetic intermediate of (+)-yahazunol (**61**), inspired by prior work of Baran’s group on divergent synthesis of meroterpenoids utilizing boronosclareolide (Scheme 5) [35]. Li’s group instead utilized compound **58**, readily available by the oxidative degradation of (−)-sclareol (**43**) [36] as the precursor to a photolabile Barton ester **59**. When the latter was irradiated at 250 W in the presence of benzoquinone, a decarboxylated coupling occurred, yielding semiquinone **60**, few steps away from the common scaffold **61**. Following this protocol, researchers managed to synthesize more than 4 g of the natural product (+)-yahazunol (**61**). (+)-Yahazunol (**61**) can be readily transformed to several members of meroterpenoids of the class, either by Friedel−Crafts reactions or common oxidative manipulations.

### Total synthesis of dysideanone B (**75**) and dysiherbol A (**79**)

**Scheme 6:**
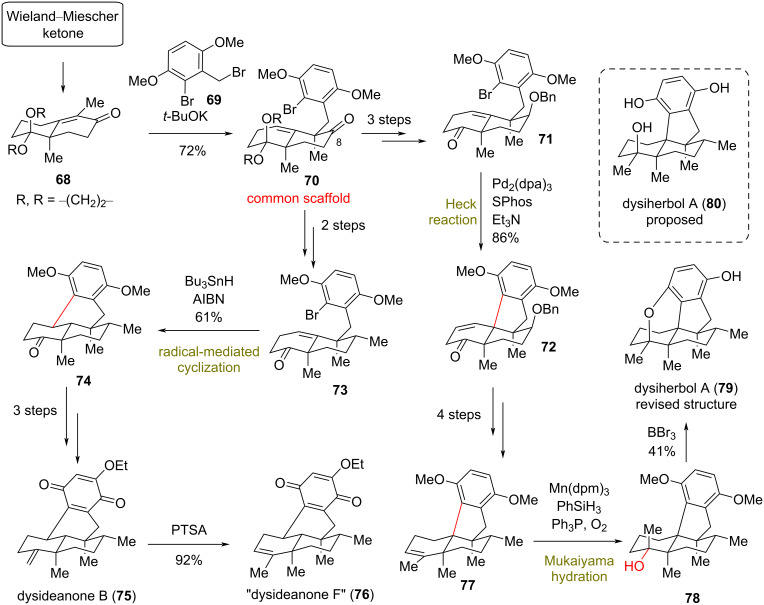
Divergent synthesis of natural products isolated from *Dysidea avara* (Lu).

**(Lu 2021)** [37]**:** Dysideanone B (**75**), isolated from the South China Sea sponge *Dysidea avara*, possesses an unprecedented 6/6/6/6-fused tetracycle with interesting anticancer properties against HeLa and HepG2 cancer cell lines (Scheme 6) [38]. The structurally similar dysiherbols **79** and **80**, bearing a 6/6/5/6-fused tetracycle instead, were reported to possess NF-kB-inhibitory activity and anticancer activity against NCI H-929 cancer cell lines (Scheme 6) [39]. In 2021 Lu’s group reported the total synthesis of members of both meroterpenoid families based on a highly chemoselective α-alkylation in the thermodynamic position of a Wieland−Miescher ketone derivative **68** with benzyl bromide **69**. Despite the challenging O- and C7-alkylations that required suppression, the desired C9-alkylation was achieved in 72% yield under thermodynamically controlled conditions (*t*-BuOK in THF at −40 °C). This coupled the terpene and the aromatic moieties present in these natural products and provided the common synthetic intermediate **70** (Scheme 6). The diverse tetracycles were accessed either via an intramolecular radical cyclization of the reduced congener **73** or through a Heck reaction of intermediate **71**. Reaction of **73** with Bu_3_SnH in the presence of AIBN as initiator provided the tetracyclic core of dysideanones. The late introduction of an ethoxy group completed the total synthesis of dysideanone B (**75**) and “dysideanone F” (**76**). Ring closure to the dysiherbol scaffold was a much more challenging task as the classic conditions of the Heck reaction to common scaffold **70** proved unsuccessful. Screening of several reaction conditions on different analogues led to the conclusion that reduction of the C8-carbonyl side and acetal deprotection to **71** are essential in order to create the 6/6/5/6-carbocycle in the presence of Pd_2_(dba)_3_, SPhos, and Et_3_N in 86% yield. Reduction of the double bond with Pd/C followed by dual Stille coupling for the introduction of two methyl groups and Mukaiyama hydration utilizing Mn(dpm)_3_ and PhSiH_3_ furnished the misassigned structure for dysiherbol A (**79**). A revised structure was finally assigned after deprotection with BBr_3_ to complete the first total synthesis of dysiherbol A (**79**).

### Total syntheses of (+)-jungermatrobrunin A (**89**) and related congeners

**Scheme 7:**
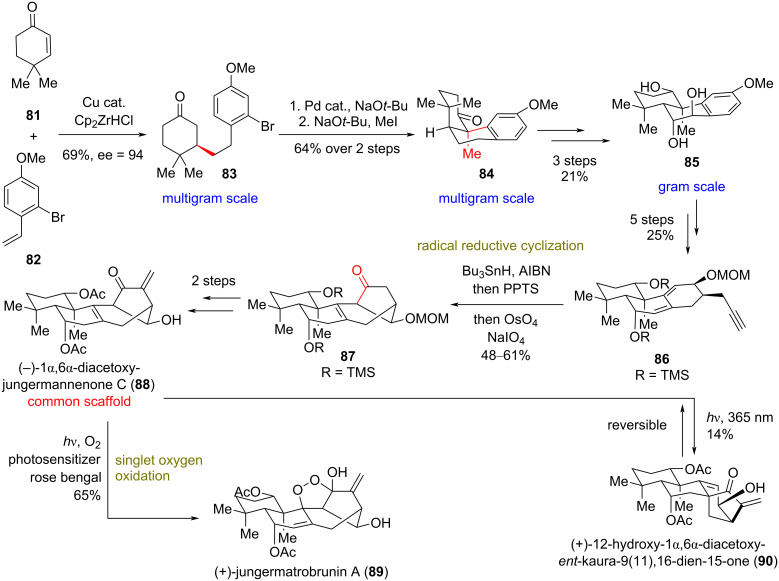
Divergent synthesis of kaurene-type terpenoids (Lei).

**(Lei 2019)** [40]**:** The *ent*-kaurane diterpenoids constitute a highly diverse class of structurally complex natural products possessing promising biological profiles, including anticancer, antifungal, and antiviral activities [41]. The highly diverse nature of the family makes a divergent synthesis extremely challenging, even for closely related members. Biosynthetically, the jungermannenone natural products have been proposed to derive from *ent*-kaurane diterpenoids through carbocationic rearrangements [42]. Jungermatrobrunin A (**89**) [43] bears a highly oxidized scaffold with a unique bicyclo[3.2.1]octene backbone and an unprecedented peroxide bridge (Scheme 7). Natural product (−)-1α,6α-diacetoxyjungermannenone C (**88**) [43] was projected by Lei’s group as the common scaffold for the divergent synthesis of this class. Finally, the closely related congener **90** [43] was envisaged to originate by a radical rearrangement of the common scaffold **88**.

Initially, Lei’s group unfolded the synthesis of **83** on a decagram scale, utilizing an asymmetric conjugate reaction of commercially available **81** and **82** using Fletcher’s protocol (94% ee) [44]. A subsequent intramolecular arylation in the α-position of the ketone of **83**, catalyzed by a Pd(II)–NHC [45], followed by methylation, provided *cis*-decalin **84** (Scheme 7). Appropriate redox modifications allowed the delivery of *trans*-decalin **85** in a gram-scale quantity. Birch reduction of the electron-rich aromatic ring, followed by propargylic addition and functional group interconversion (FGI) provided dienyne **86**. Compound **86**, under the previously developed radical reductive cyclization for 1,6-dienyne cyclization (using Bu_3_SnH and AIBN) [46], led to the construction of the key bicyclo[3.2.1]octene carbocyclic core of jungermatrobrunin, which was further elaborated to **87** in up to 61% yield, after alkene cleavage by OsO_4_ and NaIO_4_. The described reductive radical cyclization can be scaled up to 2 g without substantial decrease of the product yield. FGI, followed by methylenation provided the common scaffold **88**. Further elaboration of **88** to natural products **90** and **89** was accomplished by UV irradiation at 365 nm in MeOH and by utilizing singlet oxygen (using rose Bengal) in MeCN/pyridine, 40:1, respectively. Interestingly, irradiation at 365 nm even in the presence of photosensitizer and O_2_ failed to furnish (+)-jungermatrobrunin A (**89**), and **90** was obtained as the sole product, albeit in low yield (14%). Attempts to optimize the yield always afforded recovered **88**, hinting at a potential equilibrium between **88** and **90**.

### Total syntheses of magninoids and guignardones

**Scheme 8:**
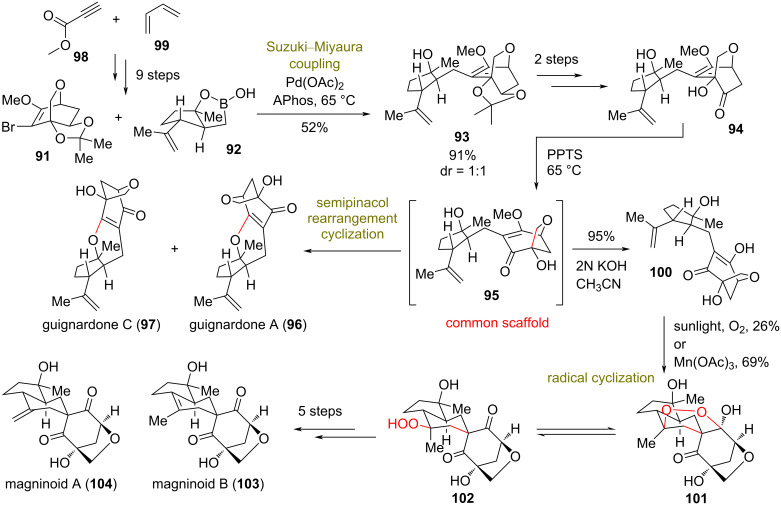
Divergent synthesis of 6-oxabicyclo[3.2.1]octane meroterpenoids (Lou).

**(Lou 2021)** [47]**:** Magninoids and guignardones are two classes of biogenetically related meroterpenoids, bearing a highly substituted cyclopentane moiety and a 6-oxabicyclo[3.2.1]octane fragment [48–49]. These classes exhibit diverse biological properties, such as potent inhibition of 11-β-hydroxysteroid dehydrogenase type I and inhibition of *Candida albicans* [48]. Although earlier syntheses have been reported recently for magninoids [50–51], Lou’s group envisioned a divergent plan based on a late-stage bioinspired semipinacol rearrangement–cyclization of common synthetic intermediates **94** and **95** (Scheme 8). Compound **94** was obtained in three steps, with the key step being the Suzuki–Miyaura coupling of appropriately functionalized precursors **91** and **92** using Romo and co-worker’s protocol [52]. Reaction of **94** under PPTS acidic conditions initiated a semipinacol rearrangement leading to **95**, followed by subsequent cyclization to natural products guignardone A (**96**) and C (**97**). This process involved 1,2-allyl migration and C–O bond formation through a semipinacol rearrangement and a cyclodehydration cascade reaction (Scheme 8).

Following the same rationale, **94** was diverted to produce **100** after basic deprotection of the nonisolated **95**. The radical oxidation of the former in the presence of dioxygen and sunlight or a catalytic amount of Mn(OAc)_3_ led to the creation of the compounds **101** and **102**. FGI, followed by the cleavage of the hydroperoxide bond and final dehydration by Burgess reagent provided the total syntheses of magninoids A (**104**) and C (**103**, Scheme 8).

### Divergent total synthesis of crinipellins

**Scheme 9:**
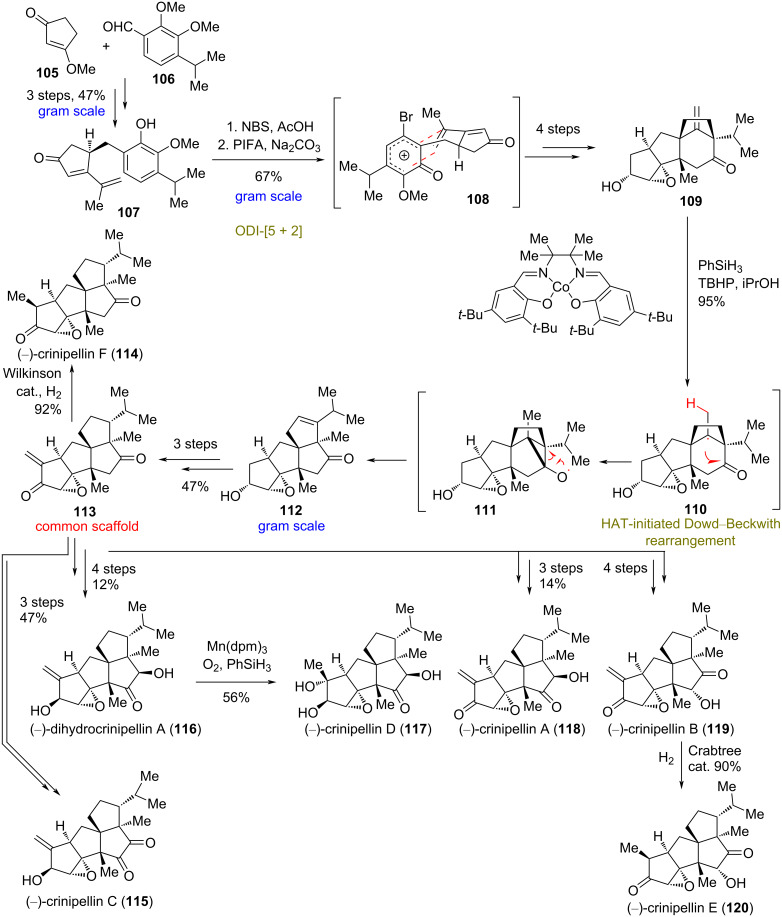
Divergent synthesis of crinipellins by radical-mediated Dowd–Backwith rearrangement (Xie and Ding).

**(Xie and Ding 2022)** [53]**:** Crinipellins are highly congested tetraquinane natural products comprising 6–10 stereogenic centers, three of which are consecutive all-carbon quaternary carbon atoms [54–56]. Preliminary biological screening of this family revealed notable antibacterial and anticancer activities due to the α-methylene lactone moiety they bear [57]. Recently, in order to synthesize the common core present in crinipellins, Xie and Ding’s groups developed an approach using an unprecedented ring distortion. Their strategy consisted of a metal-catalyzed HAT to the *exo*-Δ-alkene of the 5/5/6/5 tetracycle **109**, so as to subsequently favor a Dowd–Beckwith rearrangement [58] towards the tetraquinane skeleton of **112** (Scheme 9). The synthesis commenced with the generation of **107** from cyclopentenone **105** and aryl aldehyde **106** in a three-step sequence. An oxidative dearomatization induced a [5 + 2] cycloaddition–pinacol rearrangement of **107** to **109**, according to previous studies of the same group (Scheme 9) [59–61]. The key HAT-mediated rearrangement was realized in an impressive yield of 95% to obtain **112** on a gram-scale, when cobalt complex C6 was used in the presence of PhSiH_3_ and TBHP in isopropanol. Further modifications of **112** led to the common scaffold **113** in 47% yield, which could be readily transformed to several crinipellin natural products by chemoselective redox reactions.

### Divergent total synthesis of *Galbulimima* alkaloids

**Scheme 10:**
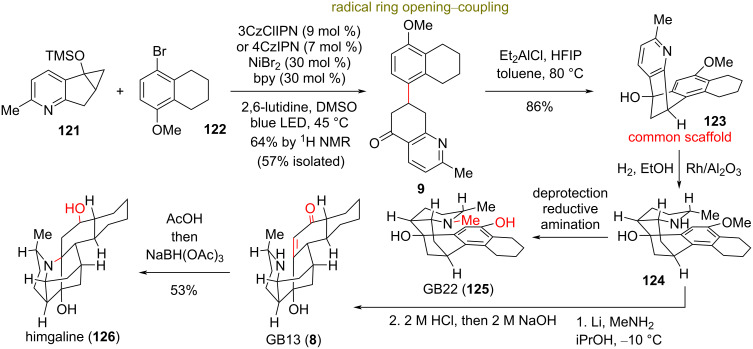
Divergent total synthesis of *Galbulimima* alkaloids (Shenvi).

**(Shenvi 2022)** [62]**:** Members of the *Galbulimima* alkaloids extracted from rainforest canopy trees were found to possess neuroactive properties, such as antagonistic activity at muscarinic receptors [63], psychotropic activity, and antiplasmodic activity [64]. Their structural diversity, consisting of different connectivities between piperidine and decalin domains, is especially difficult to be divergently accessed. Shenvi’s group recognized that an aromatic congener within this class could be traced back to aromatic common intermediate **9** (Scheme 10). Despite the simplicity, the most obvious disconnections, such as an anionic enone conjugated addition and a direct cationic Friedel–Crafts reaction failed. Highlighting the power of radical disconnection, the group thought of utilizing a β-keto carbon-centered radical to circumvent the unsuccessful Friedel–Crafts reaction. Prior reports implicated β-keto radical formation in the ring opening of siloxycyclopropanes with photoinduced electron transfer (PET) to 1,4-dicyanonaphthalene [65]. Inspired by reports on dual photoredox and Ni-catalytic cross-coupling platforms [66], the group considered a system in which a photoexcited catalyst oxidatively cleaves a siloxycyclopropane with endo selectivity [67], leading to aryl–nickel capture and reductive elimination. Thus, when substrates **121** and **122** were photoirradiated with blue LED light at 45 °C in the presence of lutidine base, 7 mol % organic photocatalyst 4CzIPN, 30 mol % NiBr_2_, and 30 mol % bpy provided 57% of **9**. Intramolecular Friedel–Crafts reaction by Et_2_AlCl and HFIP complex led to **123**, possessing the correct connectivity for the divergent synthesis of the family. Choreographically executed sequential reduction steps allowed the total synthesis of GB13 (**8**), himgaline (**126**), and GB22 (**125**) in only one third of the number of steps of prior syntheses (Scheme 10).

### Concise syntheses of eburnane alkaloids

**Scheme 11:**
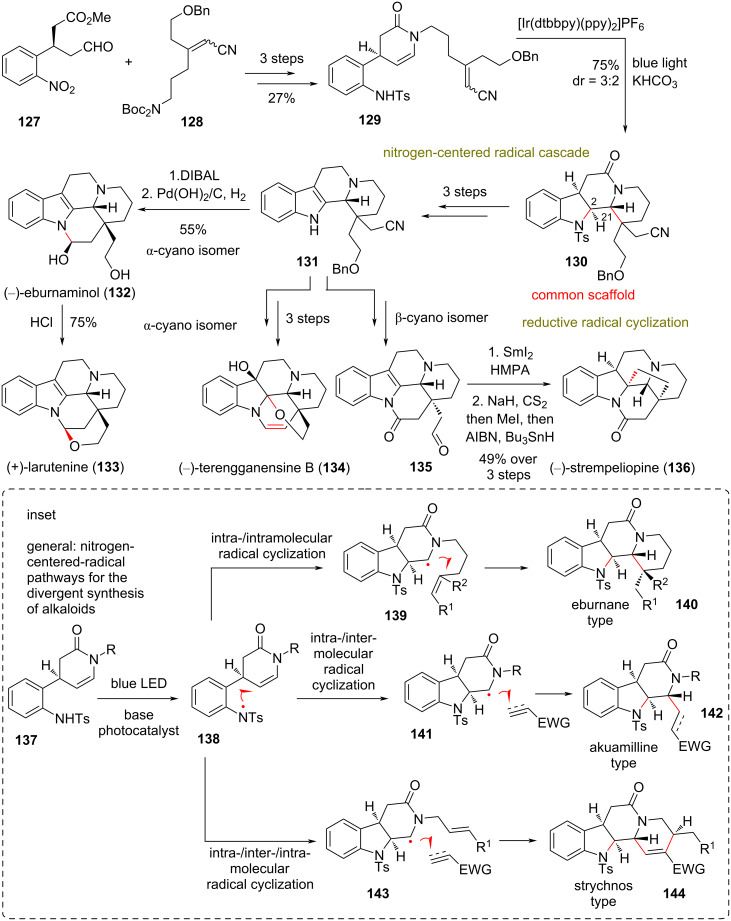
Divergent synthesis of eburnane alkaloids (Qin).

**(Qin 2018)** [68–69]**:** Eburnane indole alkaloids comprise a highly diverse class of natural products mainly distributed in Southeast Asia and China [70]. Compounds of this class are traditionally used for detoxification and as anti-inflammatory agents in Chinese medicine [71]. Qin and co-workers reported the asymmetric total syntheses of several eburnane alkaloids. Therein, they relied on one of their previous discoveries, namely a photoredox-catalytic nitrogen-centered radical cascade [72], which has resulted in the impressive collective total synthesis of 33 alkaloids of three different classes of indole natural products (please see the inset of Scheme 11 for concise representation). Specifically, this included (–)-eburnaminol (**132**), (+)-larutenine (**133**), (–)-terengganensine B (**134**), and (–)-strempeliopine (**136**), as well as the asymmetric formal total synthesis of (–)-terengganensine A (not shown, Scheme 11). The requisite common synthetic intermediate **129** for the cascade was accessed by an acid-promoted condensation of chiral aldehyde **127** and Boc-protected amine **128**, followed by zinc reduction of the nitro group and subsequent protection of the amine by a tosyl group in 27% overall yield. Irradiating **129** with blue light at 30 W in the presence of 1 mol % of [Ir(dtbbpy)(ppy)_2_]PF_6_ and 5 equiv of KHCO_3_ in THF resulted in the radical formation of the tetracyclic core of **130** in 75% yield as a mixture of two diastereoisomers (dr = 3:2) that were both used to access natural products. Impressively, the protocol allowed the installation of three rings and the stereoselective introduction of chiral centers at C2 and C21 for the final targets. With regard to the mechanism, it is hypothesized that it commences with the formation of a nitrogen-centered radical. The carbon radical **139** is then formed after the aforementioned nitrogen radical attacks the enamide group. The α-amide positioning is theorized to improve drastically the radical stability, nucleophilicity, and selectivity of **139** [73]. Furnishing of the common scaffold **130** can be carried out via an attack of intermediates of this type (e.g., **139**) on Michael acceptors. Tosyl group deprotection of **130**, followed by selenium anhydride oxidation and catalytic reduction of the amide using Wilkinson’s catalyst provided diastereoisomeric indole **131**. Careful manipulation of the nitrile and alcohol side chains allowed selective cyclizations to the nitrogen atom of the indole core to conclude the total syntheses of **132**–**134**. Samarium diiodide-mediated reductive cyclization of aldehyde **135**, obtained also from **131**, provided the pentacyclic core of (−)-strempeliopine (**136**) as a single diastereoisomer in 65% yield. Then, Barton’s radical deoxygenation resulted in the total synthesis of **136**. Further, FGI of both diastereoisomers of **130** allowed the formal synthesis of (−)-terengganensine A (not shown) under the same divergent plan (Scheme 11).

### Divergent total synthesis of (−)-pseudocopsinine (**149**) and (−)-minovincinine (**150**)

**Scheme 12:**
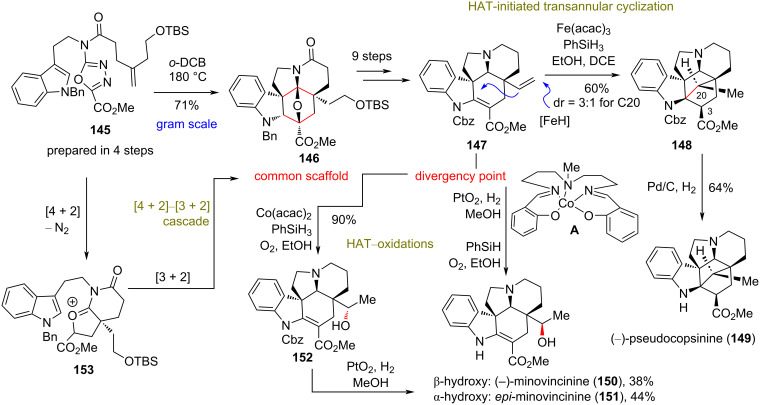
Divergent synthesis of *Aspidosperma* alkaloids (Boger).

**(Boger 2020)** [74]**:** (−)-Pseudocopsinine (**149**) was isolated from *Vinca erecta*, with a structure related to the *Aspidosperma* alkaloids, containing an additional C20–C2 bond (Scheme 12) [75]. In 2020, Boger’s group reported the first total synthesis of (−)-pseudocopsinine (**149**) and (−)-minovincinine (**150**) from a common intermediate **146**, featuring a late-stage HAT strategy to assemble the highly congested carbocyclic core of these natural products (Scheme 12). Based on earlier studies of the group on the total synthesis of vinblastine and related natural congeners [76], the authors realized that a late-stage formation of the C20–C2 bond would be highly strategic to provide the greatest simplification to these targets. The *Aspidosperma* skeleton **146** of both natural products was accessed in a single step from **145** through a scalable tandem [4 + 2]–[3 + 2] cascade in 74–84% yield in gram-scale quantities, known from previous studies [77]. Compound **145** was readily prepared in four steps from *N*-benzyltryptamine and 4-(2-*t*-butyldimethylsilyloxy)pent-4-enoic acid, requiring only two purification steps [77]. FGI of **146** led to (−)-enantiomer **147**, which serves as the radical point od divergence of this plan. HAT-initiated transannular free-radical cyclization of (−)-enantiomer **147** according to Baran’s protocols [78] provided the benzyl-protected (−)-pseudocopsinine **148** in 60% yield, when **147** was treated with PhSiH_3_ in the presence of Fe(acac)_3_. Notably, the reaction provided a diastereoselectivity of 3:1 for the formation of the C20-stereocenter and exclusive formation of the C3-center. Key to this success is the low level of Fe(III)–H generation, thus minimizing intermediate radical reduction. The observed diastereoselectivity can be rationalized by referring to earlier mechanistic studies [79]. The same (−)-configured intermediate **147** was utilized in a HAT-initiated oxidation to access (−)-minovincinine (**150**) in 38% yield after deprotection (Scheme 12). Interestingly, the classic Mukaiyama conditions using Co(acac)_2_ with PhSiH_3_ provided compound **152** as the only isomer which, upon reduction, led to the exclusive formation of the compound *epi*-minovincinine (**151**). Replacement of Co(acac)_2_ with Co complex **A** suppressed the formation of **152** and provided the desired **150** and the isomer as an almost 1:1 mixture.

### Syntheses of (−)-FR901483 (**160**) and (+)-TAN1251C (**162**)

**Scheme 13:**
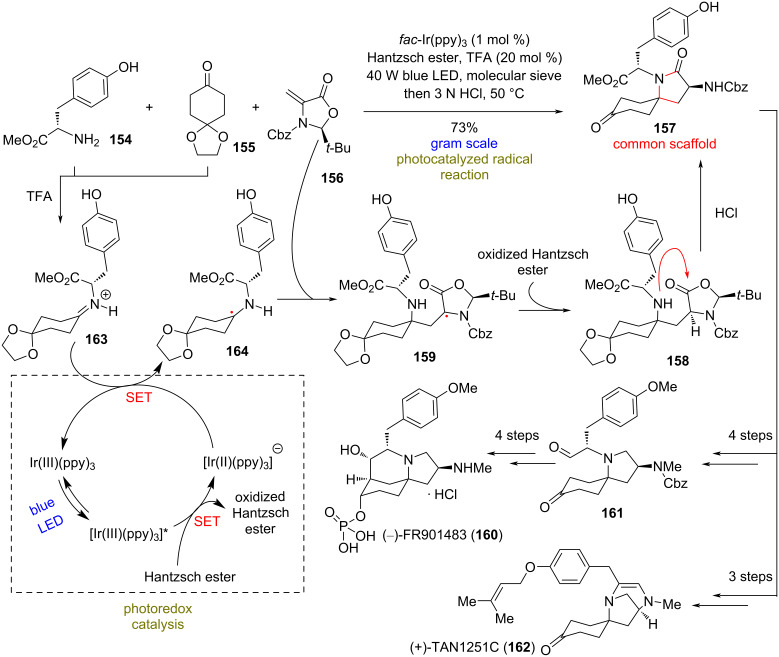
Photoredox based synthesis of (−)-FR901483 (**160**) and (+)-TAN1251C (**162**, Gaunt).

**(Gaunt 2020)** [80]**:** Nitrogen-spirocyclic natural products consist a common class of important pharmaceutical candidates. FR901483, which was isolated from the fermentation broth of *Cladobotryum sp.* No. 11231, exhibits impressively potent immunosuppressant activity. This has resulted in extensive synthetic efforts towards the compound, in order to meet the needs for the supply as a potential therapeutic for the treatment of arthritis, Crohn’s disease, and organ transplant rejection [81]. The TAN1251 natural products, on the other hand, show potent activity as muscarinic antagonists, with potential applications as antispasmodic and antiulcer agents [82]. Despite the synthetic efforts on these natural products [83–86], in 2020, Gaunt’s group recognized a novel common synthetic intermediate in the structure of spirolactam **157** to access the family (Scheme 13). To synthesize it, they conjectured that a tyrosine amino acid, a cyclohexadione derivative, and a nonracemic dehydroalanine derivative could be effectively combined to build the core structure, using an already known iridium-photocatalyzed radical reaction [87]. Indeed, when ʟ-tyrosine methyl ester (**154**), 1,4-cyclohexanedione monoethylene acetal (**155**), and dehydroalanine derivative **156** were allowed to react in the presence of TFA, molecular sieves, 1 mol % of *fac*-Ir(ppy)_3_, and Hantzsch ester under blue LED irradiation at 40 W, this resulted in the formation of spirolactam **157** in 73% yield (Scheme 13). The reaction is estimated to take place initially with the one electron reduction to α-amino radical **164**. This step is thought to be facilitated after TFA protonates the formed imine. Afterwards, radical addition of **164** to **156**, generates an α-carbonyl species. A HAT from Hantzsch ester, which takes place diastereoselectively from the more accessible face, afforded the lactone **158**. Spirolactam **157** can effortlessly be produced after the cyclization of the aforementioned lactone. Redox manipulations from this point on brought about the total synthesis of (−)-FR901483 (**160**) through an aldol reaction, and an intramolecular condensation resulted in the synthesis of (+)-TAN1251C (**162**, Scheme 13).

### Divergent synthesis of bipolamine alkaloids

**Scheme 14:**
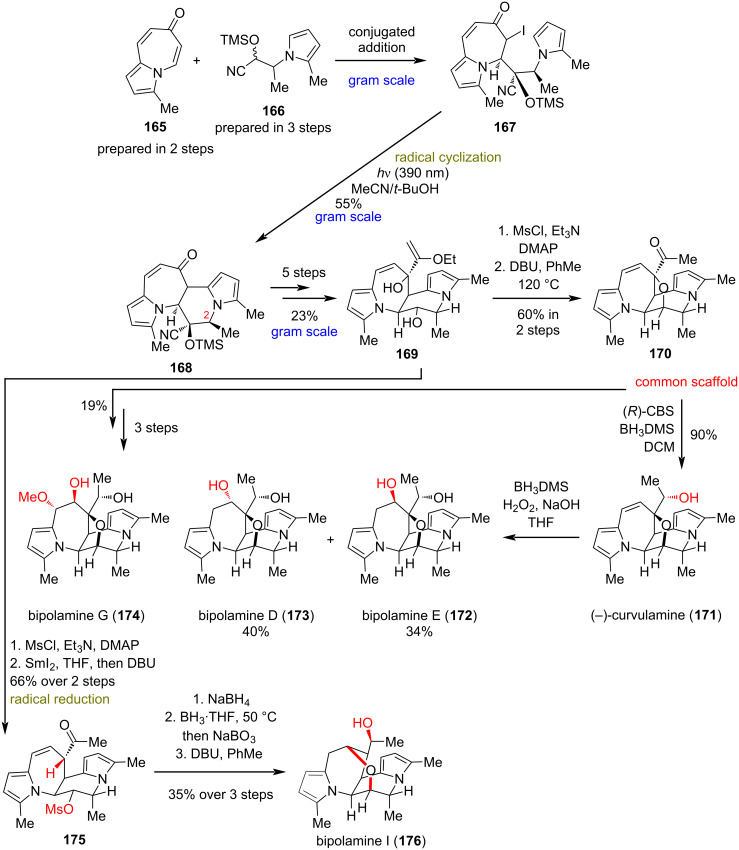
Divergent synthesis of bipolamines (Maimone).

**(Maimone 2022)** [88]**:** Bipolamines were isolated from the fungi *Curvularia sp.* IFB Z10 and *Bipolaris maydis* in 2014 and were reported to possess antibacterial activity against a small panel of both gram-positive and -negative bacteria [89–90]. Interestingly, their chemical structure bears no resemblance to recognize antibiotics and their mechanism of action remains unknown. Based on the knowledge gained from the first total synthesis of (−)-curvulamine (**171**) [91], Maimone’s group leveraged their plan for accessing several members of this class (Scheme 14). The challenge this group had to address in this particular case was the high acid sensitivity and oxidative fragility of pyrrole intermediates. As common synthetic intermediate, the group utilized compound **170**, readily available in gram-scale quantity, through a modified previously reported sequence [91]. The synthesis of the sterically constrained tetracyclic core of **170** relied initially on the photochemical radical cyclization of iodide **167** at 390 nm in the presence of NaHCO_3_ in CH_3_CN/*t*-BuOH, 5:1 to provide **168** in 55% yield (Scheme 14) [91]. Alkylation of the tetracycle, followed by epimerization of the C2 center and radical deoxygenation, or alternatively S_N_2 etherification, provided the common scaffold **170**. The latter can serve as ideal diversification point to access (−)-curvulamine (**171**) by CBS reduction, bipolamines D (**173**) and E (**172**) by additional BH_3_·DMS hydroboration, and bipolamine G (**174**) initially by dihydroxylation of the alkene moiety with osmium tetroxide, followed by acidic etherification and reduction. Finally, bipolamine I (**176**) was obtained from **169** via a samarium diiodide reduction of the mesylate, followed by sodium borohydride reduction of the ketone, hydroboration, and base-mediated cyclization.

### Flow-controlled divergent synthesis of aporphine and morphinandienone natural products

**Scheme 15:**
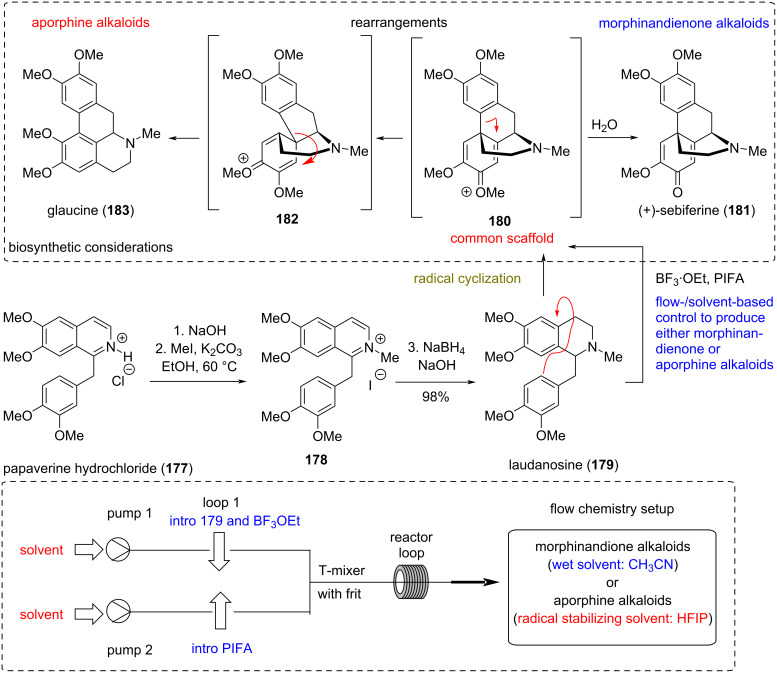
Flow chemistry divergency between aporphine and morphinandione alkaloids (Felpin).

**(Felpin 2022)** [92]**:** Reticuline-type alkaloid oxidative coupling is a well-established biosynthetic pathway that produces important pharmaceutical structures [93], such as (+)-corytuberine, (−)-codeine, (−)-morphine, (+)-sebiferine (**181**), etc., depending on the regioselectivity of the coupling (Scheme 15) [94]. During this process, two major families of natural compounds are formed, namely the aporphine and the morphinandienone alkaloids. Mimicking the selectivity of the natural process in laboratory setups commonly proves tricky, producing an irreproducible yield of isomers for both classes. Recently, Felpin’s group reported the flow-controlled divergent synthesis of aporphine and morphinandienone alkaloids based on biomimetic common scaffolds (e.g., **180**) using hypervalent iodine(III) reagents. Capitalizing on previously reported mechanistic investigations, they assumed that **180** can rearrange to glaucine (**183**) through the erythrinadienone intermediate **182**. On contrary, common scaffold **180** should hydrolyze to sebiferine-type scaffolds in the presence of water. Taking these results into account, the group exploited the ability of HFIP to stabilize the radical cation formed by PIFA and BF_3_·EtO_2_ [95–96] to selectively produce aporphine natural products, while the use of PIDA or PIFA in the presence of BF_3_·OEt or TMSOTf in wet CH_3_CN allows to diverge the synthesis to morphinandienone natural products (e.g., **181**, Scheme 15). The flow reaction was performed in a reaction coil at room temperature. Two reaction loops were used. The first one was loaded with the substrate and the second with PIFA and BF_3_·EtO_2_, while HFIP was used as the solvent. The two streams were mixed in a T-mixer, equipped with a 250 μL frit, to ensure efficient mixing. Under the optimized conditions, the method provided aporphine products in good to moderate yield, depending on the substrates used. Altering the solvent to wet CH_3_CN allowed the efficient delivery of morphindienone compounds.

### Pyrroloazocine natural products

**Scheme 16:**
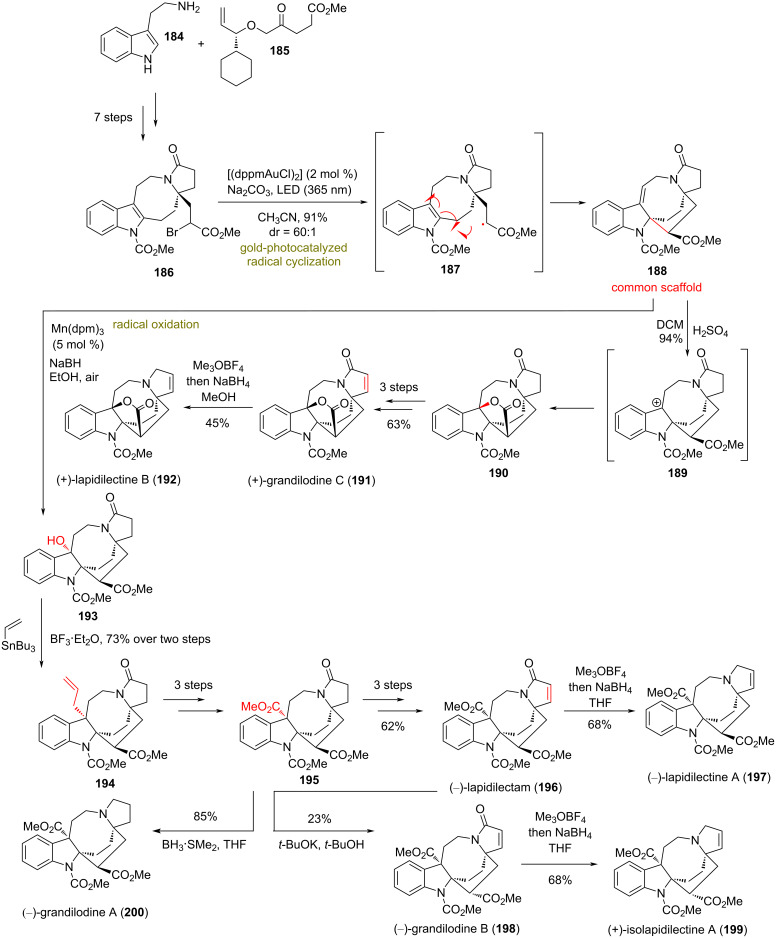
Divergent synthesis of pyrroloazocine natural products (Echavarren).

**(Echavarren 2018)** [97]**:** In 2018, Echavarren’s group reported the divergent synthesis of several pyrroloazocine alkaloids [98–100]. Preliminary biological screening indicates that members of this class are able to overcome multidrug resistance in vincristine resistant cells [98–100]. To access the common scaffold **188**, the group relied on an intramolecular gold-photocatalyzed radical-mediated cyclization of an α-keto radical to the pendant indole core, reported earlier in the total synthesis of lundurines A–C (Scheme 16) [101]. The authors postulate that photoexcitation of [(dppmAuCl)_2_] with 365 nm light serves as initiator for radical generation in the brominated position of **186**, prepared after following a 7-step sequence. The cyclization of the formed radical is 6-*exo*-*trig* and leads to the formation of a benzyl radical that is further oxidized to **188**. From this common scaffold, the group managed to access several natural products of the class, majorly by utilizing the ability of conjugated alkenes to be further oxidized, and thus producing the respective benzylic cation. Intramolecular cyclization in the cationic position under participation of the methyl ester function provided the core for (+)-grandilodine C (**191**) and (+)-lapidilectine B (**192**), while allylation of the benzylic position allowed oxidative decomposition to the core of **194** and **195**. FGI followed to complete targets **196**–**200** (Scheme 16).

### Pyrroloindoline natural products

**Scheme 17:**
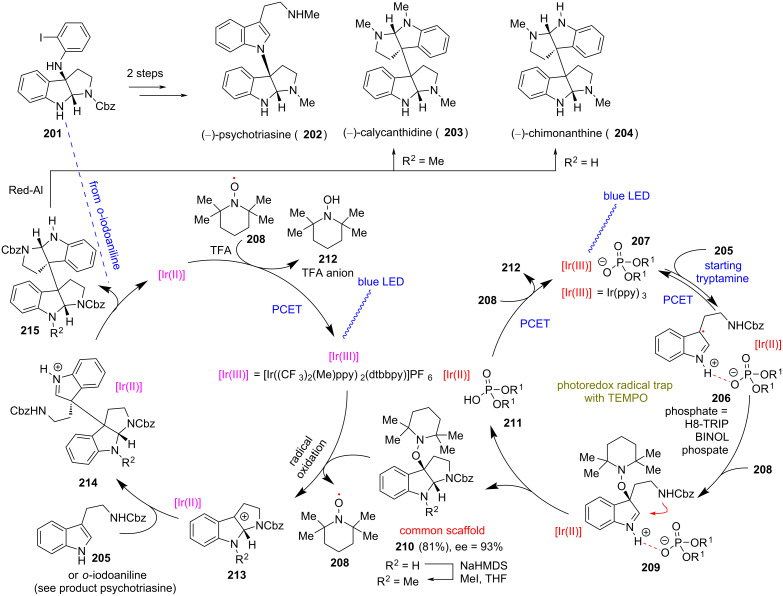
Using TEMPO to stabilize radicals for the divergent synthesis of pyrroloindoline natural products (Knowles).

**(Knowles 2018)** [102]**:** In 2018, Knowles’ group demonstrated the ability of TEMPO to act as a trap for radical cations arising from the single-electron oxidation of protected tryptamine starting materials. The utilization of a chiral phosphate base is essential for the formation of a hydrogen bond between phosphate and tryptamines, allowing the decrease of the oxidation potential. This concept was used for the synthesis of pyrroloindoline natural products (Scheme 17). Thus, upon irradiation, iridium polypyridyl photocatalyst allowed the oxidation of the phosphate complex **207** to radical cation **206**, which can be readily trapped by TEMPO, and hence stabilizing the imine and allowing cyclization with the pendant amine to form the pyrroloindoline core **210** in 81% yield and 93% ee. The latter can serve as a common scaffold to access an array of pyrroloindoline natural products but also synthetic analogues (Scheme 17). Oxidation of **210** by a second iridium photocatalyst yields benzyl cation **213**, which can undergo nucleophilic attack by tryptamine derivatives to allow the total synthesis of (−)-psychotriasine (**202**), (−)-calycanthidine (**203**), and (−)-chimonanthine (**204**).

### Synthesis of structurally diverse lignans

**Scheme 18:**
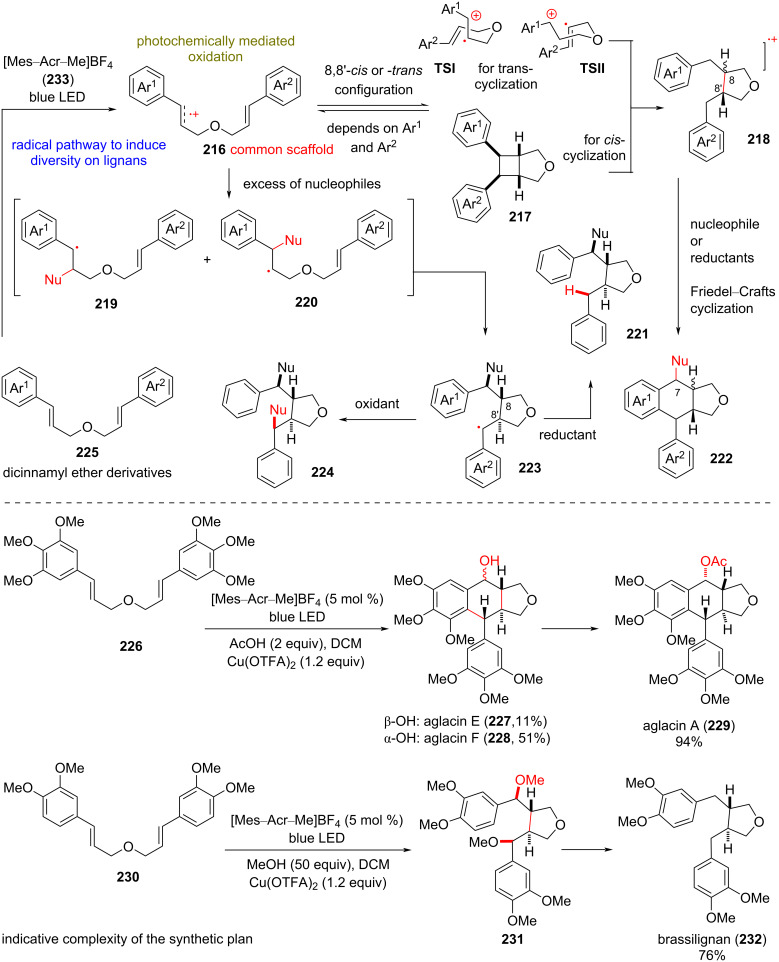
Radical pathway for preparation of lignans (Zhu).

**(Zhu, 2022)** [103]**:** Lignans are structurally diverse natural compounds generated biosynthetically by the oxidative dimerization of phenylpropanoids [104]. Despite the wide oxidative diversity, classic lignans bearing a C8–C8’ bond can be biosynthetically traced back to coniferyl alcohol (Scheme 18). Commonly, lignans possess important pharmacological properties including antimicrobial, anti-inflammatory, immunosuppressive activities, etc. [105]. At the same time, some members have been recognized as potent topoisomerase inhibitors and have been used as anticancer drugs [106]. To access the rich diversity of this class, Zhu’s group recently applied a Fukuzumi salt ([Mes–Acr–Me]BF_4_)-mediated photochemical oxidation of dicinnamyl ether derivative **225** in the presence of appropriate additives (Scheme 18). According to the postulated mechanism, the reaction is initiated by an SET of the dicinnamyl ether substrate to Fukuzumi’s salt **233**, leading to radical cation **216**. Earlier findings of the same group [107] revealed that substitution on the aryl groups is the determinant factor for either 8,8’-*cis*- or 8,8’-*trans*-cyclization to furan heterocycle cation **218**, which serves as the hypothetical common scaffold of the plan. Diverting this mechanistic route to different lignans is possible by introducing nucleophilic additives (e.g., MeOH), oxidants (e.g., Cu(OTFA)_2_), or quenchers (e.g., PhSSPh) to the reaction mixture. When monosubstitution of the aryl group is present, the formed radical cation, the product of the photooxidation of the cinnamyl ether, readily cyclizes to cyclobutene radical cation **217**. The latter cleaves the benzylic C–C bond to produce the 1,4-radical cation *cis*-**218**. On the other hand, when polysubstitution with methoxy groups is present, the cation in **216** is delocalized, inhibiting the production of cyclobutene **217**. Thus, radical cyclization according to the Beckwith–Houk model [108–109] via transition states **TSI** and **TSII** would take place, leading to the intermediate *trans*-**218**. In the presence of external nucleophiles (e.g., MeOH), the cation can be trapped, leading to substitution in the 7-position, while the radical is postulated to be oxidized to a cation, followed by a Friedel–Crafts reaction to the final product **222**. When an excess of nucleophile is employed, radical **223** is favored, leading to either monosubstitution or disubstitution with external nucleophiles, depending on the presence of oxidant or reductant in the reaction mixture. Based on this plan, Zhu’s group managed to synthesize a rich number of lignans and congeners, such as aglacin A (**229**), β-ΟΗ-aglacin E (**227**), α-ΟΗ-aglacin F (**228**), brassilignan (**232**), etc.

### Diverse synthesis of highly oxidized dibenzocyclooctadiene (DBCOD)-type lignans

**Scheme 19:**
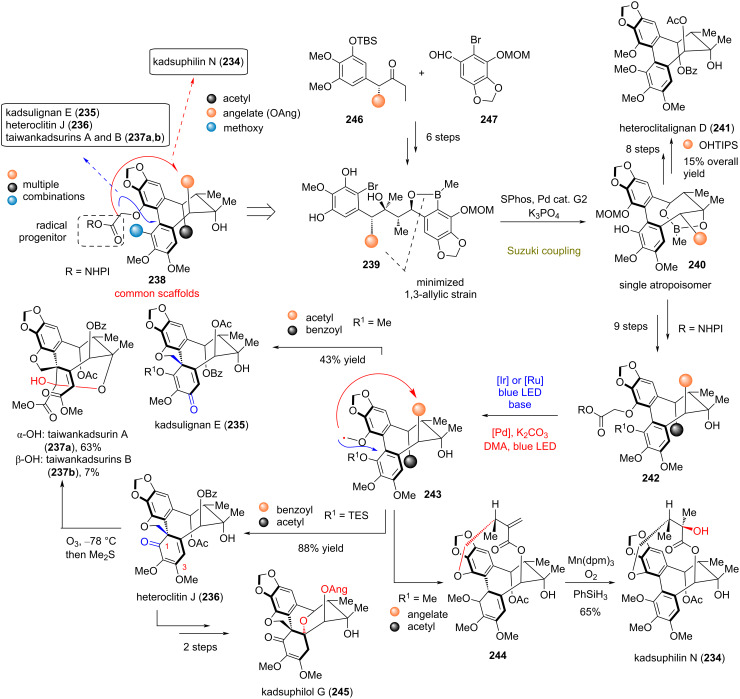
Divergent synthesis of DBCOD lignans (Lumb).

**Lumb (2021)** [110]**:** Extracts of *Schisandraceae* are rich sources of highly oxidized DBCOD lignans with interesting biological properties [111]. Designing their divergent plan on postulated biosynthetic steps, Lumb’s group managed to efficiently prepare DBCOD derivatives **238** bearing the appropriate handles for late-stage radical formation (Scheme 19). Their success relied on the strategic design of linear precursors **239**, bearing the appropriate substitution for the minimization of 1,3-allylic strain to enable Suzuki coupling for biaryl formation as a single atropisomer. The optimized conditions for this transformation utilize Buchwald’s catalyst (SPhos and Pd-based G2 precatalyst) in conjunction with K_3_PO_4_. With DBCOD bearing carboxylic acid handles at the 19-position in hands, the group proceeded with the generation of requisite radical **243** from the respective phthalimide ester under photocatalyzed conditions, either with [Ir(dtbpy)(ppy)_2_](PF_6_) or [Ru(bpy)_3_](PF_6_)_2_ in the presence of base. The reaction provided a good yield of the cyclized products kadsulignan E (**235**) and heteroclitin J (**236**) depending on the appropriate substitution of DBCODs. Selection of radical termination at the 3- and 1-positions, respectively, can be engineered by the strategic incorporation of a TES protecting group at the 1-position (see **243**) for heteroclitin J (**236**). Further treatment of heteroclitin J (**236**) with ozone provided the selective formation of taiwankadsurins A and B (**237a**,**b**) by initial oxidative cleavage of the electron-rich aromatic ring and subsequent formation of the lactole ring. Heteroclitin J (**236**) has also been transformed to kadsuphilol G (**245**) by basic deprotection of the acetyl and benzoyl groups, followed by intramolecular cyclization and angelate esterification. Also, the differently redox-active DBCOD bearing an angelate functional group enabled the synthesis of kadsuphilin N (**234**). The synthetic sequence utilizes Fu’s protocol for palladium-mediated photocatalysis (using Pd(PPh_3_)_2_Cl_2_ in combination with xantphos) [112] towards **244**, followed by Mukaiyama hydration with the aid of Mn(dpm)_3_, dioxygen, and PhSiH_3_ for the synthesis of kadsuphilin N (**234**).

## Conclusion

The utility of radical retrosynthetic disconnections in natural product synthesis is highlighted in practice, day after day, when shorter and scalable syntheses are coming into light. Combining these advantages with the power of divergent synthesis provides a yet underdeveloped strategy to address the challenges that insufficient supply of pharmaceutical leads poses, enriching the chemical libraries with natural scaffolds for biological screening. The evidenced increase of divergent radical syntheses in the last few years indicates that this approach is here to change the way chemists will practice total synthesis in the future.
